# The European tiered approach for virucidal efficacy testing – rationale for rapidly selecting disinfectants against emerging and re-emerging viral diseases

**DOI:** 10.2807/1560-7917.ES.2021.26.3.2000708

**Published:** 2021-01-21

**Authors:** Maren Eggers, Ingeborg Schwebke, Miranda Suchomel, Valerie Fotheringham, Jürgen Gebel, Bernhard Meyer, Graziella Morace, Hans Joachim Roedger, Christine Roques, Pilar Visa, Katrin Steinhauer

**Affiliations:** 1 Labor Prof Gisela Enders MVZ GbR, Stuttgart, Germany; 2 Robert Koch-Institute, Berlin, Germany; 3 Institute of Hygiene and Applied Immunology, Medical University, Vienna, Austria; 4 Evans Vanodine International PLC, Preston, United Kingdom; 5 Institute for Hygiene and Public Health, University of Bonn, Germany; 6 ECOLAB Deutschland GmbH, Monheim, Germany; 7 Istituto Superiore di Sanità, Rome, Italy; 8 Lysoform Dr. Hans Rosemann GmbH, Berlin, Germany; 9 Faculté des sciences pharmaceutiques, Paul Sabatier University, Toulouse, France; 10 Eurofins BioPharma Product Testing Spain S.L.U, Barcelona, Spain; 11 Schülke & Mayr GmbH, Norderstedt, Germany

**Keywords:** COVID-19, pandemic, European Standardisation, emerging virus, outbreak, disinfectants, hand hygiene, surface disinfection, infection prevention and control

## Abstract

When facing an emerging virus outbreak such as severe acute respiratory syndrome coronavirus 2 (SARS-CoV-2), a quick reaction time is key to control the spread. It takes time to develop antivirals and vaccines, and implement vaccination campaigns. Therefore, preventive measures such as rapid isolation of cases and identification and early quarantine of cases’ close contacts—as well as masks, physical distancing, hand hygiene, surface disinfection and air control—are crucial to reduce the risk of transmission. In this context, disinfectants and antiseptics with proven efficacy against the outbreak virus should be used. However, biocidal formulations are quite complex and may include auxiliary substances such as surfactants or emollients in addition to active substances. In order to evaluate disinfectants’ efficacy objectively, meaningful efficacy data are needed. Therefore, the European Committee for Standardisation technical committee 216 ‘Chemical disinfectants and antiseptics’ Working Group 1 (medical area) has developed standards for efficacy testing. The European tiered approach grades the virucidal efficacy in three levels, with corresponding marker test viruses. In the case of SARS-CoV-2, disinfectants with proven activity against vaccinia virus, the marker virus for the European claim ‘active against enveloped viruses’, should be used to ensure effective hygiene procedures to control the pandemic.

## Background

When facing an emerging infectious disease outbreak, a quick reaction time is crucial to control the spread. However, it takes time to develop and produce therapeutics and/or vaccines [1]. The challenge becomes even greater with a fast-spreading disease such as coronavirus disease (COVID-19) [2]. Like viruses that cause severe acute respiratory syndrome (SARS), Ebola virus disease, Middle East respiratory syndrome (MERS) and Nipah virus infection, SARS coronavirus 2 (SARS-CoV-2)—the causing agent of COVID-19—is an example of a virus that has emerged at the human-animal interface within the last two decades [2-6]. Additionally, air travel has facilitated an increasingly interconnected world with more frequent cross-border movements of people and trade goods, enabling the rapid spread of infectious diseases globally [7].

Measures are needed to control COVID-19, whether these are non-pharmaceutical, a pharmaceutical prophylactic or a pharmaceutical treatment [8,9]. Though COVID-19 pandemic activity decreased in Europe in the early summer months of 2020, a second wave began in autumn and it is possible that additional waves could occur this winter [10]. Non-pharmaceutical measures (NPM)—including rapid isolation of positively tested cases and identification and early quarantine of cases’ close contacts, as well as barriers such as masks, physical distancing [11], hand hygiene, surface disinfection and air control in concerned hospital settings—remain crucial to reduce the risk of person-to-person transmission of SARS-CoV-2 by asymptomatic, presymptomatic or symptomatic infected persons in healthcare and non-healthcare (e.g. community) settings.

The use of antiseptics and disinfectants has long been a widely accepted part of infection prevention and control to support healthcare professionals, patients and people at higher risk of serious illness with rapid and efficacious measures. In general, biocidal disinfectant or antiseptic formulations are complex and may include auxiliary substances such as surfactants or emollients in addition to active substances, which can influence the effect of the disinfectant. Therefore, recommending to use a product that contains a certain biocidal active substance is not adequate to identify suitable products.

In general, it is important to have a standardised test protocol for comparing the efficacy of disinfectant and antiseptic products [12]. In order to select antiseptics and disinfectants with proven efficacy, the European Committee for Standardisation (CEN, Comité Européen de Normalisation in French) established the technical committee (TC) 216 in 1990 for chemical disinfectants and antiseptics. The scope of this TC is to set up guidance for the standardisation of the terminology, requirements, test methods (including potential efficacy under in-use conditions), recommendations for use and labelling of chemical disinfectants and antiseptics. This committee consists of delegates from different European countries, with representatives from governments, competent authorities (i.e. public health institutes, regulatory authorities), official institutes, universities, test laboratories and manufacturers of antiseptics and disinfectants.

European standards for testing antiseptics and disinfectants are based on a tiered approach, using relevant surrogate test organisms. This helps to facilitate efficacy tests and to develop adequate practical recommendations regarding the disinfectants that can be used immediately in an ongoing outbreak, which will add confidence to the bundle of measures necessary to prevent spread of infectious diseases.

## Test principle of European standards: surrogate test viruses and tiered test approach in EN 14476

Information on the efficacy of disinfectants and antiseptics against emerging enveloped viruses such as Ebola virus (EBOV) or SARS-CoV-2 is limited because of the high biosafety level (BSL) of these viruses or because they are not available in laboratories, particularly at the beginning of an outbreak. To avoid this problem, vaccinia virus (modified vaccinia virus Ankara (MVA) or vaccinia virus strain Elstree, respectively) was introduced as a surrogate virus for the European claim ‘active against enveloped viruses’ in 2015, after in vitro studies comparing MVA with EBOV, SARS-CoV-1 and MERS-coronavirus (CoV) using povidone-iodine (PVP-I) showed that vaccinia virus was the most resilient virus [13,14]. This is in line with a publication that demonstrated in its investigation of enveloped viruses—such as Zika virus, EBOV and coronaviruses—that vaccinia is a suitable and safe surrogate when looking at the virucidal efficacy of alcoholic formulations for hand disinfectants [15]. Meanwhile, the efficacy of these disinfectants (PVP-I and alcoholic World Health Organization formulations) has also been proven against SARS-CoV-2 [16,17].

The European approach with surrogate organisms has been effective for more than 60 years against bacteria and for about 40 years against viruses, e.g. in German national standards. The choice of surrogate viruses is of great importance when establishing a standardised test. The requirements for standardised surrogate viruses are: high resistance to disinfectants and drying, combined with simple virus propagation in cell culture, availability in virus collections and a low BSL (BSL 1 and 2). For example, 30 years ago, European virucidal activity tests focused only on the most resistant virus: hepatitis A, a picornavirus that it is difficult to handle in a routine testing laboratory. The poliovirus 1 Sabin vaccine strain Lsc-2ab, another picornavirus, can easily be handled in cell culture for virus propagation and therefore became the first surrogate virus for the claim ‘virucidal activity’.

Presently, the tiered approach on the virucidal standard EN 14476 [18] consists of two parts: three different activity levels and two kinds of test methods, as shown in Table 1.

**Table 1:** Standard test methods to substantiate activity claims for medical products for hand, surface and instrument disinfection, according to EN 14885

Type of activity	Test virus	Product claim/field of application						
		Phase/step	Hygienic handrub	Hygienic handwash	Surgical handrub or handwash^a^	Surface disinfection		Instrument disinfection
						Without mechanical action	With mechanical action	
**Virucidal activity against enveloped viruses**	Vaccinia virus (strain Elstree and/or or MVA) *Enveloped*	2/1	EN 14476	EN 14476	NI	EN 14476	EN 14476	EN 14476^b^
	Vaccinia virus (strain Elstree and/or MVA) *Enveloped*	2/2	NA	NA	NI	EN 16777	Approved^c^	EN 17111 [30] (exclusively for pre-cleaning products)
**Limited spectrum of virucidal activity against enveloped viruses plus noro-, rota- and adenoviruses**	Adenovirus (adenovirus type 5, strain Adenoid 75) *Non-enveloped* Murine norovirus (MNV, strain S99) *Non-enveloped*	2/1	EN 14476	EN 14476	NI	EN 14476	EN 14476	NI
	Adenovirus (adenovirus type 5, strain Adenoid 75) *Non-enveloped* Murine norovirus (MNV, strain S99) *Non-enveloped*	2/2	prEN 17430 (exclusively murine norovirus)	Not yet approved^b^	NI	EN 16777	Not yet approved^b^	NI
**Virucidal activity**	Poliovirus type I (PV1, strain LSc-2ab) *Non-enveloped* Adenovirus (adenovirus type 5, strain Adenoid 75) *Non-enveloped* Murine norovirus (MNV, strain S99) *Non-enveloped*	2/1	EN 14476	EN 14476	NI	EN 14476	EN 14476	EN 14476 (≥ 40 °C: exclusively MVM for chemothermal disinfection)
	Poliovirus^d^ *Non-enveloped* Adenovirus (adenovirus type 5, strain Adenoid 75) *Non-enveloped* Murine norovirus (MNV, strain S99) *Non-enveloped*	2/2	prEN 17430 (exclusively murine norovirus)	Not yet approved^b^	NI	EN 16777	Not yet approved^b^	EN 17111 [30] (≥ 40 °C: exclusively MVM for chemothermal disinfection)

MNV: murine norovirus; MVA: modified vaccinia virus Ankara; MVM: minute virus of mice; NA: not applicable; NI: no intention to develop a test; PV1: poliovirus type 1.

^a^ EN 14476 is not applicable for surgical handrub and handwash products because surgical products are designed to be effective against resident microbiota and viruses are part of the transient microbiota.

^b^ No work item yet approved, but relevant standards may become available in the future.

^c^ Work item approved.

^d^ Must be tested in the corresponding suspension test; poliovirus cannot be used for surfaces, because of drying problems.

The three European virucidal activity levels take into account that enveloped viruses contain lipids and have lipophilic properties (Box). These viruses are sensitive to disinfectants that contain active substances that disrupt their lipid bilayer envelope. In contrast, non-enveloped viruses are usually hydrophilic and less susceptible to disinfectants. Thus, viruses can be grouped according to these properties (Table 2).

**Table 2:** Activity spectrum of the surrogate viruses for virucidal efficacy testing

Activity claim	Test virus	Activity spectrum (examples^a^)
**Virucidal activity against enveloped viruses**	Vaccinia virus (strain Elstree and/or MVA) *Enveloped*	**Viruses causing blood-borne infections** Hepatitis B virus (HBV), hepatitis C virus (HCV), human immunodeficiency virus (HIV) **Viruses causing respiratory infections** Human coronaviruses (e.g. SARS-CoV-2 and MERS-CoV), influenza virus A (e.g. subtype H1N1, H3N2) and B, metapneumovirus, respiratory syncytial virus (RSV) **Viruses causing other viral infections** Herpesviridae (cytomegalievirus (CVMV), herpes-simplex-virus types 1 and 2 (HSV-1, HSV-2), Epstein-Barr virus (EBV), varizella zoster virus (VZV)) Paramyxoviruses (Nipah virus, measles virus, mumps virus, rubella virus) **Viruses causing travel-associated or vector-borne infections** Bunyavirus (sandfly fever virus), dengue virus (DENV), Ebola virus (EBOV), tick-borne encephalitis virus (TBEV), Hantaan virus, Crimean-Congo hemorrhagic fever virus, Lassa virus, Marburg virus, rabies virus, West Nile virus (WNV), Yellow fever virus (YFV), Zika virus
**Limited spectrum of virucidal activity:** **enveloped viruses plus noro-, rota- and adenoviruses**	Adenovirus (adenovirus type 5, strain Adenoid 75) *Non-enveloped* Murine norovirus (MNV, strain S99) *Non-enveloped*	**Viruses mentioned above** **Viruses causing viral gastrointestinal infections** Adenovirus serotypes 40 and 41, norovirus, rotavirus **Viruses causing respiratory infections** Adenovirus serotype 7 **Viruses causing keratoconjunctivitis** Adenovirus serotypes 8, 19 and 37
**Virucidal activity:** **non-enveloped viruses and enveloped viruses**	Poliovirus type I (PV1, strain LSc-2ab) *Non-enveloped* Adenovirus (adenovirus type 5, strain Adenoid 75) *Non-enveloped* Murine norovirus (MNV, strain S99) *Non-enveloped*	**Viruses mentioned above** **Viruses causing gastrointestinal infections** Adenovirus serotypes 40 and 41, norovirus, rotavirus **Viruses causing respiratory infections** Adenovirus serotype 7 **Viruses causing keratoconjunctivitis** Adenovirus serotypes 8, 19 and 37 **Viruses causing other virus infections** Enteroviruses (Coxsackie virus, echovirus, polioviruses, rhinoviruses, enterovirus 71, Enterovirus 68 (EV-D68)) Parechoviruses (echovirus 22 and 23)
**Chemothermal disinfection ≥ 40 °C**	Murine parvorvirus (minute virus of mice (MVM), strain Crawford) *Non-enveloped*	**Textile disinfection or instrument disinfection**

MVA: modified vaccinia virus Ankara.

^a^ This list is not exhaustive.

Box **European virucidal activity levels**

• ***Virucidal activity against enveloped viruses*:** Activity against enveloped viruses (e.g. influenza (including avian influenza A (H7N9)), herpes viruses, HIV, HBV, HCV, Zika virus, Ebola virus, coronaviruses (including SARS-CoV-2 and MERS-CoV) and flaviviruses). This claim is for hand disinfection, surface disinfection and pre-cleaning products for instruments, which are combined cleaner/disinfectants.

• ***Limited spectrum virucidal activity*:** Activity against non-enveloped noroviruses, rotaviruses, adenoviruses and enveloped viruses. This claim is for hand disinfection and surface disinfection.

• ***Virucidal activity:*** Activity against viruses, including non-enveloped (e.g. polioviruses and enteroviruses, such as enterovirus 71 and enterovirus D68) and enveloped viruses. This claim is for hand disinfection, surface disinfection, instrument disinfection and textile disinfection.

HBV: hepatitis B virus; HCV: hepatitis C virus; MERS-CoV: Middle East respiratory syndrome coronavirus; SARS-CoV-2: severe acute respiratory syndrome coronavirus 2.

## Test methods to prove efficacy of a disinfectant

The CEN/TC 216 requires a stepwise procedure for efficacy testing of disinfectants, i.e. first a test in suspension (EN phase 2, step 1), followed by a test simulating practical conditions in the laboratory (EN phase 2, step 2). This tiered approach, including organic soiling, is described in the framework standard EN 14885 [19]. The quantitative suspension test EN 14476 is a phase 2, step 1 test [18] and is the first tier to evaluate the performance of the disinfectant in terms of concentration and contact time ratio. The EN 14476 demands a 4 log reduction (deactivating 99.99%) of the respective test viruses as the minimum measure of efficacy. In addition, activity tests have to be performed in compliance with adequate quality assurance systems and according to valid test procedures, as described in the relevant European standard(s) [19]. In recent years, tests simulating practical conditions have been developed for surface, instrument and hand disinfectants that take into account the application of a specific disinfectant under evaluation.

### Phase 2, step 2 tests reflecting close to real-life conditions

#### Surface disinfection

Viruses may persist on surfaces for several days or even months [20] and can be transferred directly from contaminated surfaces to susceptible individuals [21]. Therefore, the disinfection of surfaces frequently touched by patients and staff such as door handles, faucets and railings plays an important role in the prevention and control of viral outbreaks in healthcare settings. Disinfectants must be tested for their efficacy under standardised, close to real-life conditions to ensure that they are able to inactivate viruses. This approach has long been standardised according to European principles for testing the bactericidal activity of chemical disinfectants [22,23]. Bactericidal disinfectants are tested stepwise according to European test principles, starting with a suspension test (EN phase 2, step 1) and then a quantitative non-porous carrier test simulating practical conditions (EN phase 2, step 2) [18]. It is also possible for the activity of virucidal products intended for surface disinfection to be tested under practical conditions (phase 2, step 2) according to the EN 16777 standard [24,25].

#### Handrub disinfection

In order to further improve our knowledge of the time/concentration relationships of handrubs, taking into consideration practical conditions for use in clinical surroundings, more practical testing (phase 2, step 2 tests) are necessary, such as the EN 1500 for evaluating the bactericidal efficacies of hygienic handrubs [26]. In such tests, the bactericidal efficacy of products is compared with that of a reference procedure on hands of volunteers artificially contaminated with *Escherichia coli* K12. Since most emerging viruses are enveloped viruses, a relevant surrogate for products with activity against enveloped viruses should be sought. More than 30 years ago, tests with vaccinia virus were performed on the fingertips of volunteers [27]; however, today this can no longer be justified ethically due to safety concerns. Thus, there is currently no approved surrogate enveloped virus available for testing hand hygiene products that is harmless and can be used in studies with volunteers. Until a suitable assay with an appropriate surrogate can be proposed, only data of the EN 14476 suspension test (phase 2, step 1) is available. However, there is a preliminary phase 2, step 2 standard prEN 17430 [28] that employs the murine norovirus strain S99, a non-enveloped and non-pathogenic RNA virus to simulate practical conditions for virucidal hygienic handrub products; this is harmonised with the EN 1500 standard. Being more resistant to chemicals than enveloped viruses, the murine norovirus can be considered a suitable surrogate to support the claims ‘limited spectrum virucidal activity’ and ‘virucidal activity’ (if all criteria have been met in a phase 2, step 1 test) based on the tiered concept of CEN/TC 216 [29].

## Conclusions

Disinfectants and antiseptics are part of the bundle of measures necessary to achieve infection prevention and control in healthcare settings and the community. The European tiered approach of grading the virucidal efficacy as ‘virucidal activity against enveloped viruses’, ‘limited spectrum of virucidal activity’ and ‘virucidal activity’ helps to select the most appropriate claim for emerging or re-emerging virus outbreaks, with a proven concentration and contact time. This leads to the correct choice of test method to determine the appropriate use, concentration and contact time of antiseptics and disinfectants.

The standards outlined in Table 1 are subject to mutual recognition in the European Union and replace previous national standards. Furthermore, the standardised test protocol based on the use of surrogate viruses and two methodical steps also helps to compare formulations in an objective way, thus providing proven efficacy to the end user.
